# Predicting Critical Care Nurses' Intention to Use Physical Restraints in Intubated Patients: A Structural Equation Model

**DOI:** 10.1155/2023/3286312

**Published:** 2023-07-13

**Authors:** Yajun Ma, Nianqi Cui, Xiaoli Li, Jiayi Li, Yuping Zhang, Yonggang Liu, Jingfen Jin

**Affiliations:** ^1^Nursing Department, The Second Affiliated Hospital of Zhejiang University School of Medicine (SAHZU), Hangzhou, China; ^2^School of Nursing, Kunming Medical University, Kunming, China; ^3^First Clinic, Kunming Medical University School and Hospital of Stomatology, Kunming, China; ^4^Department of Critical Care Medicine, Yan'An Hospital of Kunming City, Kunming, China; ^5^Department of Critical Care Medicine, First Affiliated Hospital of Kunming Medical University, Kunming, China; ^6^Key Laboratory of the Diagnosis and Treatment of Severe Trauma and Burn of Zhejiang Province, Hangzhou, China; ^7^Changxing Branch Hospital of SAHZU, Huzhou, China

## Abstract

**Aims:**

To identify the factors influencing critical care nurses' intention of physical restraint in intubated patients.

**Background:**

Physical restraint reduction has been advocated by many international institutions, nurses are the main physical restraint decision-makers, and it is critical to identify the factors influencing physical restraint intention from nurses' perspective.

**Methods:**

A cross-sectional study was conducted among critical care nurses in China from February 2022 to March 2022.

**Results:**

The model showed a good model fit (*χ*^2^/d*f* = 2.57, RMSEA = 0.07, GFI = 0.94, CFI = 0.89, and AGFI = 0.90). Attitude (*β* = 0.29, *p* < 0.05), subjective norm (*β* = 0.25, *p* < 0.05), and perceived behavioral control (*β* = 0.32, *p* < 0.001) directly influenced the intention to use physical restraint in intubated patients. Ethical conflict (*β* = 0.04, *p* < 0.05) indirectly influenced the intention.

**Conclusions:**

The study revealed that ethical conflict, attitude, subjective norm, and perceived behavioral control were positive predictors of physical restraint intention among intubated patients from nurses' perspectives. *Implications for Nursing Management*. This provides a theoretical perspective to develop effective interventions to reduce physical restraints in critical care settings. Nursing managers should enhance ethical education and physical restraint knowledge and skill training.

## 1. Introduction

Critically ill patients often rely on a series of life support equipment and invasive treatments (e.g., endotracheal intubation and central venous catheterization) throughout the intensive care unit (ICU) stay, which may result in agitation, pain, and delirium [1]. Unfortunately, these disturbing symptoms could lead to adverse events including self-extubation and medical device removal [2, 3], compromising patient safety seriously.

Physical restraint (PR) is commonly perceived as a routine solution to avoid self-extubation empirically [2, 4]. PR is defined as “any action or procedure that prevents a person's free body movement to a position of choice and/or normal access to his/her body by the use of any method, attached or adjacent to a person's body that he/she cannot control or remove easily” [5]. According to a prospective observational study, mechanical ventilation was an independent risk factor for PR use [6]. Compared to nonintubated patients, PR use is more pervasive (35.8% in Jodan [7] and 75% in Japan [8]) among mechanically ventilated patients in critical care settings [2, 6, 9].

However, there is growing evidence identifying the association between PR and deleterious effects both physical and psychological [10–12]. Besides, PR itself is regarded as a violation of autonomy and dignity [13, 14]. Furthermore, it remains to be a controversial issue whether PR could ensure patients' safety effectively, and previous studies [12, 15, 16] have indicated that PR may exacerbate unplanned extubation and medical device removal conversely.

Due to the abovementioned potential hazards, how to facilitate minimizing programs of PR has become a global issue, and PR reduction has been advocated by many international institutions including the Registered Nurses' Association of Ontario (RNAO), the American Nurses Association (ANA), and the British Association of Critical Care Nurses (BACCN). As we know that critical care nurses are the main decision-makers in PR practice, so a profound understanding of nurses' intention to use PR is an essential prerequisite in the development of PR reduction.

Previous studies have revealed the process and nurses' experience of decision-making of PR. Shen et al. [17] proposed a four-stage process of PR including perceptions of risks, hesitation, implementation, and reflection. The safety of patients and staff is seen as the core element in the process. In addition, it is conventionally believed that the application of PR is an infallible guarantee of security. Patient safety was being prioritized in the clinical context but at the expense of ignoring human rights including autonomy and dignity [18].

Despite the existence of numerous studies concerning PR practices, most of them are focused on describing the experience of nurses, knowledge, attitude, and practice of PR, lacking a theoretical framework to analyze the intention of PR. The theory of planned behavior (TPB), developed by Ajzen [19], is a widely recognized psychological framework applied in various fields, including healthcare. In the context of critical care, the TPB has been previously used in several studies to understand and predict human behaviors related to healthcare practices. For instance, O'Boyle et al. [20] used the TPB-based theoretical model to explain the self-reported and observed handwashing behavior of critical care and postcritical care nurses. Another study conducted by Tanguay et al. [21] aimed to examine the factors that influence nurses' intentions to practice oral care with intubated clients in intensive care settings using the TPB. These research contexts indicate that the TPB can assist in enhancing our understanding of the decision-making processes within the fields of intensive care and offer guidance on how to improve their practical behaviors. Besides, the rationale for applying Ajzen's TPB specifically in critical care settings lies in its ability to capture the complexity of nurses' intention formation processes from three dimensions, including attitudes, subjective norms, and perceived behavioral control. Critical care settings are characterized by high-stress levels, time constraints, and life-threatening situations where quick decision-making is required. In such circumstances, understanding the determinants of nurses' intentions towards PR becomes crucial as it directly impacts patient safety and quality of care. By taking into account these various dimensions of intention formation, the TPB model provides a new perspective in understanding the decision-making process of PR and patient care management through the lens of critical care nurses. It states that an individual's behavior is determined by his or her intention, and the behavioral intention is determined by three main dimensions: (1) attitude (the extent to which an individual has a favorable or unfavorable evaluation of the behavior), (2) subjective norm (SN perceived social pressure to perform or not to perform the behavior), and (3) perceived control of the behavior (PBC perceived ease or difficulty when performing the behavior) [19]. These factors are highly relevant in critical care nursing, where the decision to use PR is influenced by a complex interplay of individual beliefs, professional guidelines, and organizational culture. Considering these factors together makes it possible to predict the likelihood of an individual performing a specific behavior. And Via-Clavero et al. [22] developed the Physical Restraint Theory of Planned Behavior (PR-TPB) based on the TPB. Besides, from critical care nurses' perspectives, ethical conflict is regarded as an undeniable difficulty when practicing PR because the conflict between maintaining patients' safety and violating patients' autonomy and dignity often places nurses in awkward predicaments [23, 24]. Though critical care nurses have realized the adverse effects of PR, they have no choice but to use it to ensure patient safety.

Thus, we aimed to investigate the effects of TPB constructs (attitude, SN, and PBC) and ethical conflict on physical restraint intention in this study. The research question of this study is as follows: to what extent can the TPB constructs and ethical conflict predict ICU nurses' intention to use physical restraint in intubated patients?

**Figure 1:**
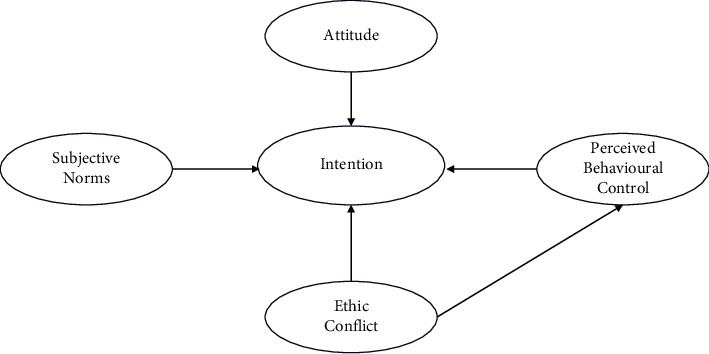
Hypothetical model.

What is new in our study is that ethical conflict was introduced as a predictor of PBC because ethical conflict is regarded as difficulty in the PR decision process. In previous studies, we found that ethical dilemma, an essential factor influencing PR practice and nurses, have reflected on the experiences of ethical dilemmas due to violations of nonmaleficence and beneficence [14]. Thus, the proposed framework is illustrated in Figure 1.

## 2. Methods

### 2.1. Design

A cross-sectional survey was conducted among critical care nurses in China from February to March 2022. In this study, structural equation modeling (SEM) was applied to establish models to predict critical care nurses' intention to use PR in intubated patients. Integrating the conceptual framework of the Theory of Planned Behavior and ethical conflict, the hypothetical model is shown in Figure 1.

### 2.2. Sample and Setting

The typical method of SEM sample size is based on the general rule of 10 : 10 observations per indicator [25–27]. In this study, it was calculated by the following equation: (4 + 2 + 3 + 3 + 19) *∗* 10 = 310 while according to Hair [28], the minimum acceptable sample size should be 300 for a model with seven or fewer constructs and factor loadings larger than or equal to 0.45. To obtain more statistically robust results, the target sample size was selected as 310 after considering both two calculation approaches. And a total of 313 critical care nurses were included in this study ultimately. Participants were recruited from critical care units covering Yunnan, Chongqing, Zhejiang, and Guangdong provinces in China via convenience sampling. The inclusion criteria of participants were (1) registered nurses who worked in intensive care units and (2) voluntary participation and informed consent to this survey. And the intern nurses were excluded.

### 2.3. Data Collection

Data were collected using a self-report questionnaire composed of three parts: (1) the Physical Restraint Theory of Planned Behavior (PR-TPB), (2) the ethical conflict in nursing questionnaire-critical care version (ECNQ-CCV), and (3) demographic information form. The data collection included two stages, Stage 1: three critical care nurses who were not involved in this study were invited to fill out the pretested questionnaires to eliminate any ambiguous or incomprehensible expressions and estimate the required time. Based on the pretest feedback, some inappropriate expressions were revised and significant words were marked. Stage 2: the researchers contacted and explained the aim of the study to the head nurses of each department and sent the revised questionnaires to the participants via an online web-based platform (https://www.wjx.cn/vm/h7e0uOF.aspx). With the assistance of the head nurses, the eligible participants were identified and organized to fill out the questionnaires. At the same time, participants were provided with a phone number to ask any questions about the study. And all of the participants were instructed to complete the questionnaire voluntarily and anonymously. To ensure the quality of returned questionnaires, the questionnaires were set as follows: (1) the questionnaire began with a concise introduction about the purpose of the study and the notes for items needing more attention were in bold and marked in red. (2) To avoid missing responses, all items were set as required questions in the submitting process and the platform will send alarms automatically if there are any missing questions. (3) To prevent repeated participation, each IP address was limited to filling out the questionnaire once only. A total of 441 questionnaires were distributed, and 316 were returned (response rate: 71.7%). In addition, 3 questionnaires with response time less than 3 minutes were excluded, and 313 valid questionnaires were selected ultimately (*n* = 313).

### 2.4. Instruments

#### 2.4.1. The Physical Restraint Theory of Planned Behavior (PR-TPB)

The PR-TPB [22] consists of the following 4 subscales: (1) attitude, (2) subjective norm, (3) perceived behavioral control, and (4) intention. All the answering formats were 7-point Likert scales ranging from 1 to 7. In this study, attitude, subjective norm, and perceived behavioral control were measured by corresponding subscales, respectively. The attitude was measured using 4 items with opposite adjectives (unsafe/safe, unnecessary/necessary, harmful/beneficial, and unacceptable/acceptable) placed on the poles of a 7-point Likert scale. The total score ranges from 4 to 28. Subjective norm was measured by 2 items describing the social pressure by the individual perceived from the working team when performing physical restraint. The score of each item is rated from 1 (strongly disagree) to 7 (strongly agree). Perceived behavioral control was measured with 3 items reflecting self-efficacy and controllability toward applying physical restraint in intubated patients. Participants rated each item from 1 (strongly disagree) to 7 (strongly agree). The intention was evaluated by 3 scenarios in ICU settings, rated from 1 (in no case) to 7 (in all cases).

Cronbach's *α* of each construct ranges from 0.6 to 0.88 [22]. Total Cronbach's *α* was 0.766 in this study.

#### 2.4.2. Ethical Conflict in Nursing Questionnaire-Critical Care Version (ECNQ-CCV)

ECNQ-CCV [29] includes 19 scenarios that may produce ethical conflict among critical care nurses, and each scenario contains three questions to measure ethical conflict: “frequency,” “degree of intensity,” and “type.” Frequency is measured with a 6-point Likert scale ranging from 0 (never) to 5 (at least once a week). The degree of intensity is measured with a 5-point Likert scale ranging from 1 (no problem at all) to 5 (highly problematic) and the type of ethical conflict is measured by six categories.

In the current study, ethical conflict was measured by the index of exposure to ethical conflict (IEEC). IEEC was a concept developed to reflect levels of exposure to ethical conflict, which multiplies the frequency and degree of intensity of each scenario with a range of 0 to 25. The total score of IEEC ranges from 0 to 475, with a higher score indicating higher levels of ethical conflict.

The instrument was tested for validity and reliability among 205 critical care nurses in Spain, which reported Cronbach's *α* of 0.882. The Chinese version of ECNQ-CCV has been validated and found to have good reliability (Cronbach's *α* = 0.902 and McDonald's *ω* 0.903) and validity [30]. Cronbach's *α* was 0.923 in this study.

#### 2.4.3. Demographic Information Form

The demographic information form included 7 questions about gender, age, work year, job title, education, training in physical restraint, and training in ethics.

### 2.5. Ethical Considerations

Ethical approval was obtained from the Second Affiliated Hospital Zhejiang University School of Medicine (SAHZU, no. 2020131). All the participations in this survey were voluntary and anonymous, and a completed questionnaire was recognized as informed consent. Participants were informed about the authorship and purpose of the research and were told that all data would remain anonymous and confidential.

### 2.6. Data Analysis

IBM SPSS Statistics version 25 software and IBM SPSS AMOS version 24 software were used in the analysis of the study. Categorical variables were described by frequency and percentage, and continuous variables were described by using means and standard deviations. The structural equation model was composed of two major elements: the measurement model and the structural model. Step one: confirmatory factor analysis (CFA) was performed to assess the reliability of the measurement model. And the correlations of constructs were calculated using Pearson's correlation coefficient. Step two: A structural model was constructed, and the model fitness was measured by the chi-square test/degree of freedom (*χ*^2^/d*f*) <3, the comparative fit index (CFI) >0.9, the goodness of fit index (GFI) >0.9, the normed fit index (NFI) >0.9, and the root mean squared error of approximation (RMSEA) <0.08 [31, 32]. The maximum likelihood (ML) estimation was used as the parameter estimation method to find the best-fitting model because skewness and kurtosis of the involved variables were within the acceptable range (absolute value of skewness <3 and absolute value of kurtosis <10), satisfying the assumption of normality [33]. And direct effects and indirect effects of the constructs were calculated by bootstrap estimates. Besides, a two-sided*p* value of 0.05 was set for statistical significance.

## 3. Results

### 3.1. Sample Characteristics

313 critical care nurses participated in the survey, and the characteristics of participants are listed in Table 1. The mean age and work year of the participants were 30.44 (SD = 6.21) and 7.42 (SD = 6.00) years, respectively; 13.7% were male and 86.3% were female. In the aspect of job title, 74.5% held a junior title, 23.6% with an intermediate title, and 1.9% with a senior title. 82.7% of participants had a bachelor's degree. Over 70% of the participants had received training in physical restraint and nursing ethics.

### 3.2. Structural Equation Modeling

#### 3.2.1. Measurement Model

In the process of confirmatory factor analysis, reliability was assessed for the measurement model.  Table 2 provides an overview of the factor loadings and composite reliability of constructs. The reliability of the measurement model was evaluated by factor loadings, composite reliability (CR), and Cronbach's alpha. The factor loadings of items varied from 0.46 to 0.84, meeting the threshold of 0.45 [34]. The values of composite reliability were above 0.5 (the criteria of CR [35]), suggesting stable composite reliability. Cronbach's alpha for all items was 0.74, which was higher than the 0.7 threshold. The preceding data confirmed the measurement model's acceptable reliability.  Table 3 shows the correlations, the mean score, and the standard deviation of each construct. Attitude (*r* = 0.26, *p* < 0.01), SN (*r* = 0.27, *p* < 0.01), and PBC (*r* = 0.29, *p* < 0.01) were positively associated with intention. IEEC was positively associated with SN (*r* = 0.12, *p* < 0.05) and PBC (*r* = 0.13, *p* < 0.05).

And the goodness of fit index of the measurement model was *χ*^2^/d*f* = 1.34 (<3), RMSEA = 0.03 (<0.08), GFI = 0.97 (>0.90), CFI = 0.98 (>0.90), and AGFI = 0.94 (>0.90). All the goodness of fit indexes indicated a satisfactory model.

#### 3.2.2. Structural Model

**Figure 2:**
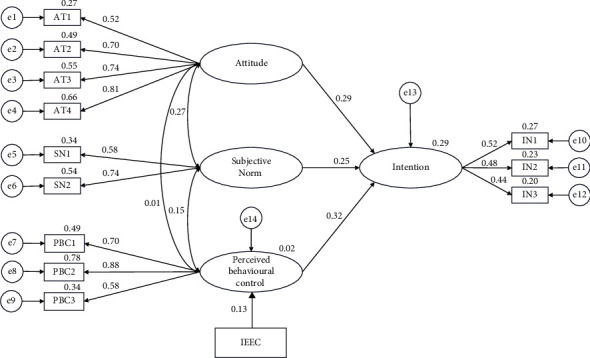
Structural model.

The final structural model is shown in Figure 2. The model was assessed by the following goodness of fit indices (*χ*^2^/d*f* = 2.57, RMSEA = 0.07, GFI = 0.94, and AGFI = 0.90); these indexes indicated a satisfactory model fit. The standardized direct and indirect path coefficients of the model are presented in Table 4. The results revealed the fact that attitude (*β* = 0.29, *p* < 0.05), subjective norm (*β* = 0.25, *p* < 0.05), and perceived behavioral control (*β* = 0.32, *p* < 0.001) had a direct effect on the intention to apply PR in intubated patients. And the index of exposure to ethical conflict has a direct effect on perceived behavioral control (*β* = 0.13, *p* < 0.05). At the same time, IEEC had an indirect effect on intention (*β* = 0.04, *p* < 0.05) via perceived behavioral control. All the paths were significant at the level of 0.05. All the variables accounted for 29% of the variance in intention to use PR in intubated patients (*R*^2^ = 0.29).

## 4. Discussion

The structural equation model revealed that ethical conflict, attitude, subjective norm, and perceived behavioral control were significant predictors of PR intention in intubated patients. Besides, this research provides a theoretical basis and new perspectives for follow-up research in the field of developing PR guidelines [36, 37].

Ethical conflict seems to be a common issue in the process of PR decisions due to the complexity of scenarios in critical care settings. As the previous study reported, nearly one-third of the nurses have confronted with ethical dilemmas in the process of physical restraints [14]. According to the results of this study, there was a positive association between ethical conflict and the intention to use PR in intubated patients, revealing that when exposed to a higher level of ethical conflict, ICU nurses are more likely to apply PR in intubated patients. The phenomenon might be explained by the flowing reasons: (1) uncertainty and depressing feelings often come with ethical conflict, and PRs may be a means to ease ethical conflict and cope with frustrating feelings. Some nurses have noted that the application of PR provides an inner sense of security and relieves the pressure of maintaining patients' safety [23]. (2) Despite violating human rights and restrictions on bodies, the security of patients is always regarded as the priority. Nurses may rationalize the implementation of PR by informing themselves that it is inevitable for security reasons. (3) Furthermore, long-term exposure to a high level of ethical conflicts may lead to a more indifferent attitude towards patients' human rights, thus turning to PR thoughtlessly. And in this study, we found out that nearly one-third of the nurses have not received ethical education. As for clinicians and policy-makers, this finding emphasized the need to cover ethical education and continued education programs among critical care nurses. At the same time, we noticed that part of nurses may be confronted with depressing moral conflict and the hospital managers need to take staff's ethical conflict into consideration and provide clinical nurses a stage to release their inner emotional burden. In the future, the effective way to identify and relieve the ethical conflict in the PR decision is needed to be explored.

In the current study, the attitude has a positive effect on the intention to use PR in intubated patients, which means the more favorable the attitude, the stronger the intention to perform the behaviors. The mean attitude was 25.32 of 28, approximately 90% of the total score, indicating a favorable attitude towards the application of PR in intubated patients among critical care nurses. Consistent with the previous study [2], such a positive attitude towards PR in intubated patients may be associated with the empirical belief that PR could prevent self-extubation and medical device removal regardless of its deleterious effects. Thus, from this perspective, more systematic and comprehensive education and training of PR are essential in reconstructing nurses' perceptions and attitudes concerning physical restriction. Several cross-sectional studies in Turkey [38], Jordan [39], and China [40] have also pointed out inadequate education and knowledge of PR. Unfortunately, despite being aware of the harmful effects of PR, some nurses are still likely to apply PR in intubated patients out of the responsibility to protect patients' safety or for a lack of other effective alternative methods. Thus, in this way, the concept of minimizing PR is needed to be advocated in critical care settings among all the medical staff.

Subjective norm has a positive effect on the intention to apply PR in intubated patients, and the mean score was more than twice as much as in Spain [41], indicating the high level of perceived social pressure to perform PRs in China compared to Spain. A qualitative study [24] has shown that PR was regarded as a routine practice in the workplace norm, and physical restraints in intubated patients were engrained in the security culture of critical care settings. Nurses, as safeguard to critically ill patients, face the burden of responsibility and pressure from the workplace. The practice of PR may be driven by expectations of the workplace other than the clinical guidelines and critical thinking of nurses. Consequently, how to reestablish evidence-based PR guidelines in Chinese hospitals and stimulate self-reflection thinking patterns are crucial issues to be examined in the development of PR reduction.

Regarding perceived behavioral control, it is the strongest positive predictor of the intention in this study. This finding infers that those nurses with higher self-efficacy and controllability toward PR are more inclined in applying PR in intubated patients. In general, senior nurses should be more proficient in PR and have higher PBC scores. However, in this study, we found an interesting thing that nurses who worked for 0–4 years had higher PBC scores than those who worked for more than 12 years. Similar to a previous study, Perez et al. [24] also found that novice nurses are more likely to use PR in intubated patients compared to senior nurses, which means there is an evident gap between the self-evaluation and the true PBC. Lacking comprehensive understanding and formal education of PR, novice nurses may overestimate their controllability of PR, thus resulting in excessive PR intention in intubated patients. In addition, due to the burden of ensuring patients' safety and a lack of other alternative methods, novice nurses are compelled to use PR. This showed that we should focus on the novice nurses' PR education, and the critical care units could assign senior nurses to guide the novice nurses in clinical PR practice.

This study has some limitations. First, the study was conducted among critical nurses in four provinces of China, the generalizability of our conclusion to other populations might be considered with caution. Another limitation lies in the fact that the TPB permits valid predictions solely when the behavior is entirely governed by volitional control, but some external factors may constrain nurses' ability to exercise full control over their PR use (e.g., patient acuity and availability of alternative interventions). Furthermore, it may not fully capture the dynamic and context-dependent nature of critical care nursing practice because it focuses on rational decision-making and the assumption of stable preferences.

In addition, the cross-sectional study cannot reflect the changing process of PR intention, and a longitudinal investigation design is needed in the future.

## 5. Conclusion

To our knowledge, this is the first study to predict critical care nurses' PR intention in intubated patients using a structural equation model. This study revealed that ethical conflict, attitude, subjective norm, and perceived behavioral control are positive predictors of PR intention in intubated patients.

## 6. Implications for Nursing Management

The present findings provide a novel theoretical standpoint for examining PR intentions within critical care environments. To mitigate PR utilization in critical care nursing, it is vital to implement a holistic approach that encompasses not only ongoing education and training on physical restraint and ethical considerations but also organizational management aspects that could affect nurses' attitudes, intentions, and actions. This may involve evaluating existing work resources, infrastructural conditions, occupation-related framework conditions, and disseminating the concept of PR reduction in clinical contexts. Moreover, it is essential to account for the accessibility of technologically sophisticated equipment. In addition, recognizing the potential impact of nursing management in promoting alternatives to PR, such as nonpharmacological approaches and patient-centered care strategies, is of utmost importance. By contemplating these wider contextual elements and fostering a more intricate comprehension of the factors influencing PR application in critical care nursing, valuable insights can be gained for the development of efficacious interventions aimed at enhancing patient safety and care quality.

## Figures and Tables

**Table 1:** Characteristics of the sample.

	*n*	(%)
*Gender*		
Male	43	13.7
Female	270	86.3

*Age 30.44 (SD* *=* *6.21)*		
20–25	74	23.6
26–30	117	37.4
31–35	62	19.8
36–40	44	14.1
>40	16	5.1

*Work year 7.42 (SD* *=* *6.00)*		
0–4	114	36.4
4–8	93	29.7
8–12	54	17.3
12–16	19	6.1
>16	33	10.5

*Job title*		
Junior	233	74.5
Intermediate	74	23.6
Senior	6	1.9

*Education*		
College	45	14.4
Bachelor	259	82.7
Master or above	9	2.9

*Training in physical restraint*		
Yes	225	71.9
No	88	28.1

*Training in ethic*		
Yes	229	73.2
No	84	26.8

*n *=* *313.

**Table 2:** The standardized factor loading and composite reliability.

Construct	Item	Unstd	SE	*Z*	*p*	Std	CR
Attitude	AT1	1.00				0.52	0.79
	AT2	1.07	0.13	8.05	^*∗∗∗*^	0.70	
	AT3	1.64	0.20	8.26	^*∗∗∗*^	0.74	
	AT4	1.28	0.15	8.48	^*∗∗∗*^	0.82	

SN	SN1	1.00				0.67	0.60
	SN2	0.93	0.13	6.92	^*∗∗∗*^	0.64	

PBC	PBC1	1.00				0.74	0.77
	PBC2	1.02	0.09	11.24	^*∗∗∗*^	0.84	
	PBC3	1.01	0.11	9.29	^*∗∗∗*^	0.59	

Intention	IN1	1.00				0.53	0.50
	IN2	1.03	0.22	4.77	^*∗∗∗*^	0.51	
	IN3	0.79	0.17	4.59	^*∗∗∗*^	0.46	

Unstd: unstandardized factor load; SE: standard error; *Z*: regression weight estimate; ^*∗∗∗*^*P* < 0.001; Std: standardized factor load; CR: composite reliability; SN: subjective norm; PBC: perceived behavioral control.

**Table 3:** Correlations, means, and standard deviations of constructs.

	1	2	3	4	5	Min–max	Mean	SD
(1) Attitude	1					4–28	25.32	2.92
(2) SN	0.17^*∗∗*^	1				2–14	10.40	2.82
(3) PBC	0.14^*∗*^	0.47^*∗∗*^	1			3–21	13.81	4.32
(4) Intention	0.26^*∗∗*^	0.27^*∗∗*^	0.29^*∗∗*^	1		3–21	16.59	3.39
(5) IEEC	−0.02	0.12^*∗*^	0.13^*∗*^	0.06	1	0–475	66.44	62.83

^*∗*^ *P* < 0.05^*∗∗*^*P* < 0.01; SD: standard deviation; SN: subjective norm; PBC: perceived behavioral control; IEEC: Index of Exposure to Ethical Conflict.

**Table 4:** Standardized path coefficients.

Path	*β*	*p*
Attitude-intention	0.29	0.005
Subjective norm-intention	0.25	0.025
Perceived behavioral control-intention	0.32	^*∗∗∗*^
IEEC-perceived behavioral control	0.13	0.038
IEEC-intention	0.04	0.017

^*∗∗∗*^ *P* < 0.001; IEEC: Index of Exposure to Ethical Conflict.

## Data Availability

The datasets used and/or analyzed during the current study are available from the corresponding authors on reasonable request.
